# The Potential of iPSCs for the Treatment of Premature Aging Disorders

**DOI:** 10.3390/ijms18112350

**Published:** 2017-11-07

**Authors:** Claudia Compagnucci, Enrico Bertini

**Affiliations:** Department of Neuroscience, Unit of Neuromuscular and Neurodegenerative Diseases, Children’s Research Hospital Bambino Gesù, IRCCS, 00146 Rome, Italy; enricosilvio.bertini@opbg.net

**Keywords:** induced pluripotent stem cells, premature aging syndromes, cell senescence, lamins, stem cell metabolism, mitochondria, nucleoskeleton, CRISPR/Cas9 technology

## Abstract

Premature aging disorders including Hutchinson-Gilford progeria syndrome (HGPS) and Werner syndrome, are a group of rare monogenic diseases leading to reduced lifespan of the patients. Importantly, these disorders mimic several features of physiological aging. Despite the interest on the study of these diseases, the underlying biological mechanisms remain unknown and no treatment is available. Recent studies on HGPS (due to mutations of the *LMNA* gene encoding for the nucleoskeletal proteins lamin A/C) have reported disruptions in cellular and molecular mechanisms modulating genomic stability and stem cell populations, thus giving the nuclear lamina a relevant function in nuclear organization, epigenetic regulation and in the maintenance of the stem cell pool. In this context, modeling premature aging with induced pluripotent stem cells (iPSCs) offers the possibility to study these disorders during self-renewal and differentiation into relevant cell types. iPSCs generated by cellular reprogramming from adult somatic cells allows researchers to understand pathophysiological mechanisms and enables the performance of drug screenings. Moreover, the recent development of precision genome editing offers the possibility to study the complex mechanisms underlying senescence and the possibility to correct disease phenotypes, paving the way for future therapeutic interventions.

## 1. Premature Aging Disorders

Premature aging disorders, also known as progeric disorders, include two rare genetic conditions, Hutchinson-Gilford progeria syndrome (HGPS) and Werner syndrome (WS). HGPS is a (autosomal dominant) genetic condition (1 in 4 million newborns are diagnosed with this condition) characterized by the rapid appearance of age-associated phenotypes beginning during childhood, in particular, premature atherosclerosis and degeneration of vascular smooth muscle cells [1]. The phenotypes associated with HGPS include alopecia, aged-skin, joint abnormalities, atherosclerosis, loss of subcutaneous fat, heart disease but not disruption of intellectual development. HGPS is caused by mutations (c.1824C > T) in *LMNA* gene, this mutation activates a cryptic splicing site and leads to the synthesis of the protein progerin, characterized by: truncation and persistence of a farnesyl residue at its C-terminal end. The mutation in the *LMNA* gene, which encodes a protein of the nucleoskeleton, causes an improperly processed version of the lamin A protein (called progerin) that accumulates in the cells and affects the nuclear architecture and cellular function. Unfortunately, there are no diagnostic tests or treatments for this progressive and fatal disease, which leads to death at a mean age of 12 from myocardial infarction or cerebrovascular accident [1,2,3]. Interestingly, progerin levels increase gradually during physiological aging and, whether this is due to (1) spontaneous mutations in *LMNA*, (2) epigenetic modifications or (3) abnormal farnesylation, it is still not fully known [4]. Other mutations in the *LMNA* gene lead to other rare disorders, including: Emery-Dreyfuss muscular dystrophy type 2; Charcot-Marie-Tooth disorder type 2B1; mandibuloacral dysplasia; limb girdle muscular dystrophy type 1B; the Dunningan type of familial partial lipodystrophy; and a familial form of dilated cardiomyopathy. Similarly, WS is a rare autosomal recessive disorder (affecting 1 in 1 million individuals) caused by mutations in the *WRN* gene, which encodes for the werner protein, involved in DNA replication during cell division. Pathogenic mutations in the *WRN* gene often cause the production of an abnormally short, nonfunctional werner protein. The disease is characterized by the appearance of multiple features of aging beginning in early adulthood [5]. Moreover, WS patients are characterized by an increased incidence of neoplasia [6]. For this reason, Werner’s syndrome has been classified into the group of chromosome instability syndromes [6]. Several studies have demonstrated the rejuvenation of the disease phenotypes following cellular reprogramming of somatic cells of patients into induced pluripotent stem cells (iPSCs) [7,8]. Werner syndrome iPSCs present a rescue of telomere function by reprogramming and several groups are investigating their epigenetic status, focusing on the accelerated epigenetic age. However, the extent to which this alteration is a cause or effect of WS disease phenotypes remains unknown and only studies involving the careful monitoring of iPSCs reprogramming and differentiation may lead to useful information to understand and treat this disease [9,10,11]. The most common causes of death for WS patients is cancer and atherosclerosis in patients between 30–50 years of age. Since the relationship between cancer and iPSCs is still unclear, we believe that further basic studies are necessary before using iPSCs for modeling and study of cancer-related diseases. For this reason, we decided to focus our attention on HGPS, where the incidence of cancer is decreased when compared to the non-HGPS population. 

Encouraging results coming from murine model of HGPS suggest a stem cell-based etiology of the HGPS and thus support the use of the iPSCs technology, as this offers the unique chance to study 1) the proliferating stem cells, 2) the differentiating and 3) the differentiated cells. In fact, a specific mouse model for HGPS has been generated (*Lmna^L530P^*^/*L530P*^) by [12] and the data obtained indicate that homozygous *Lmna^L530P^*^/*L530P*^ muscle myoblasts and fibroblasts differentiate easily into adipocytes, suggesting that defects in the nuclear lamina may be unable to preserve the state of terminal differentiation and drive the cells toward de-differentiation and re-differentiation into other cell types. These results are in line with the hypothesis that, since the progeroid syndromes affect many tissues, alterations of the nucleoskeleton affect primarily the ability and balance to self-renew and/or differentiation within the stem cell pool. In particular, Prolla suggested that the exhaustion of progenitor cells might explain the reduced regenerative ability of tissues with high cell turnover [13]. Halaschek-Wiener and Brooks-Wilson also propose that this hypothesis can explain the premature aging phenotype, and the fact that the tissues with the highest mechanical stress (i.e., blood vessels and joints) or those required to support continuous growth (i.e., hair follicles) are the tissues that degenerate in HGPS patients [14]. A stem cell-based etiology of the HGPS is also supported by the recent experimental work of Liu et al. [15], where a link between *sirtuin 1* (*SIRT1*) longevity pathway and progeria is demonstrated and resveratrol (a potential SIRT1 activator) alleviates progeroid features in affected cells [15]. In fact, the high expression levels of *SIRT1* in stem cells and its critical role in maintaining stem cell self-renewal and function, suggest that resveratrol may rescue the progeroid phenotype acting on the stem cell in *Zmpste24*^−/−^ mice, given that it does not significantly rescue senescence in somatic cells [15]. Despite this hypothesis and other animal models, robust biological data on premature aging is still lacking. A promising advancement has been performed in the study by Osorio et al. [16], as they generated a genetically modified mouse carrying the same HGPS mutation and manifesting the main clinical phenotype of human HGPS. Importantly, Osorio et al. [16] developed an antisense morpholino-based therapy that prevents the altered splicing of *Lmna*, therefore reducing the accumulation of progerin and its derived nuclear defects. The results obtained suggest that an amelioration of the progeroid phenotype occurs, thus supporting a promising route toward the development of an efficacious therapy for human patients. 

## 2. The Necessity to Develop a Good Human Model for Progeroid Syndromes

A human derived cell model recapitulating progeroid syndromes would be necessary in order to improve our understanding of the disease and to test possible therapies. For a long time, the difficulty to directly access the affected tissues has represented a strong limitation in studying the pathogenesis of premature aging because of the heterogeneity of the tissues affected (i.e., muscular, adipose, skin). Recently, this has been circumvented by the revolutionary iPSC technology. iPSCs are stem cells reprogrammed from adult somatic cells such as skin fibroblasts [17]. Interestingly, both aging and HGPS have been linked robustly to nuclear lamina dysfunction, therefore, we consider that the disruption of some biological mechanisms may be a common feature of aging and HGPS. Following these considerations, we moved on to investigate the mitochondrial features of aging and neuronal differentiation on iPSCs [18], and we decided to apply this methodology to unveil unknown aspects of nuclear lamina/mitochondrial status and disease phenotypes in aged-iPSCs [19]. Since HGPS is considered a disorder linked to defective stem cell properties (i.e., [20]), exploration by modeling iPSCs derived from affected patients seems a viable option. In fact, it has been suggested that A-type lamins can function as nuclear signaling receptors, maybe relevant for the maintenance of the stem cell pool [21]. Studies determining whether stem cells can age and defining the features of stem cells in self-renewal and differentiation are still needed. For this reason, we started to work on iPSCs obtained from healthy individuals with the aim to fully understand the iPSCs biology of aging, before considering to work on iPSCs obtained from individuals with *LMNA* mutations (affected by HGPS in particular). 

## 3. Basic Science Related to Induced Pluripotent Stem Cells (iPSCs) and Aging

iPSC-technology has allowed ‘in vitro disease modeling’ of many diseases and this technology is particularly useful to investigate pathologies where many different cell types are affected. Unfortunately, the mechanisms of iPSC aging are unknown, and for this reason we have previously investigated some features of stem cell aging and determined features that efficiently measure stem cell age in vitro [18]. Moreover, the ability to recreate the correct stem cell niche in vitro is lacking and this hinders: (1) studying the biology of stem cells (i.e., iPSCs), (2) using this technology to model human diseases, and (3) expanding them for cell therapy. Therefore, it is necessary to deepen our understanding of the molecular determinants placed in the local niche and on the mechanisms controlling self-renewal versus differentiation. Understanding iPSC reprogramming has been at the center of many studies and it is has been demonstrated that iPSCs retain a rejuvenated state able to bypass cellular aging [22]. Interestingly, Rivera-Torres et al. [23] documented a marked downregulation of mitochondrial oxydative phosphorylation proteins and a clear, mitochondrial dysfunction in HGPS cells and prompting these authors to suggest that mitochondrial dysfunction contributes to premature organ decline and aging in HGPS [23]. In addition to this, a recent study documented an increased fraction of swollen and fragmented mitochondria, together with a marked reduction in mitochondrial mobility in HGPS fibroblasts [24]. Importantly, mitochondria and nuclear envelope have both aging-related alterations and they allow cells to sense and respond to the extracellular environment, but at present very few groups have been involved in understanding the iPSC biology of aging and how these mechanisms integrate into the complexity of the stem cell niche. Understanding whether there are biological differences between young and aged iPSCs function and their in vitro environment, their mitochondrial status and their nuclear envelope integrity, is not only relevant to scientists engaged in the study of iPSC disease models and aging disorders, but more importantly, has the potential impact to accelerate the development of therapeutic applications with iPSCs. It is commonly accepted that iPSCs can be maintained indefinitely in culture [25,26], but our studies on young and aged iPSCs demonstrate the opposite. In fact, iPSCs kept in culture for prolonged time (one year) present an altered mitochondrial number and functionality and, their neuronal differentiation potential is impaired [18]. We investigated the number and functionality of young (y)-and aged (a)-iPSC mitochondria with the aim to characterize aspects of iPSC biology that are still lacking but necessary before considering iPSCs as a model system to unveil the biology of progeroid syndromes. Additionally, we studied the nuclear integrity and mechanical stability of the nuclear envelope, which are often disrupted in aging, in y- and a-iPSCs. We find that induced aging in iPSCs is characterized by altered nuclear architecture, imbalance between nucleoskeletal components (lamin A/C-prelamin isoforms, lamin B1, emerin, nesprin-2), leading to impaired nucleo-cytoplasmic megakaryoblastic leukemia (translocation) 1 or MKL1 shuttling, actin polymerization defects, mitochondrial dysfunctions, SIRT7 downregulation and hyperactivation status of NF-κBp65 transcription factor [19]. We show that aged-iPSCs present aging-related features of the nuclear envelope similarly observed in premature-aging syndromes (i.e., HGPS) and during cell senescence [19]. With these findings, the iPSC system emerges as a potential model to investigate premature aging syndromes of genetic origin as it allows studying the characteristics of stem cells during self-renewal and during cell differentiation. The iPSCs can, in fact, be guided (with specific protocols) to differentiate into different cell types (i.e., neurons, hepatocytes, astrocytes, adipocytes, smooth muscle cells, cardiomyocytes, etc.). 

## 4. Therapeutic Implications

### 4.1. Does iPSC-Based Research Offer a Good Model to Study Premature Aging?

iPSCs from fibroblasts obtained from patients have been developed to model HGPS. Interestingly, HGPS-iPSCs lack the nucleoskeletal and epigenetic alterations normally associated with premature ageing and do not accumulate progerin [27]. Moreover, upon differentiation of HGPS-iPSCs, progerin and its aging associated phenotype are observed. In fact, differentiating HGPS-iPSCs into smooth muscle cells make the prematurely senescent phenotypes evident [27].

The iPSC model was also used by Nissan et al. [28] to investigate the molecular underpinnings of the absence of neuronal degeneration in HGPS patients. Their results support the hypothesis that the restricted expression miR-9 in neural cells is the reason why this specific cell lineage is protected from PROGERIN accumulation in HGPS [29].

Colman and colleagues also used HGPS iPSCs to obtain and study neural progenitors, endothelial cells, fibroblasts, vascular smooth muscle cells (VSMCs), and mesenchymal stem cells (MSCs). Interestingly, progerin levels were high in MSCs, VSMCs, and fibroblasts, and these cell-types also presented augmented DNA damage and nuclear abnormalities. Both HGPS-MSC and-VSMC viability was severely altered by hypoxia in vitro and in vivo. Since MSCs are placed in niches with low oxygen under physiological conditions, they affirm that, in HGPS, this leads to additional depletion of the MSC pool responsible for replacing differentiated cells that died because of progerin toxicity [30].

The work by Liu et al. [27], Nissan et al. [29] and Zhang et al. [30] demonstrates the usefulness of an iPSC-based disease models of HGPS. These models enrich information from existing approaches using animal and cell models, and allow the study of tissue specific expression of the disease-associated gene at endogenous levels. Importantly, the demonstration that cell specific differentiation of HGPS-iPSCs can yield phenotypes that recapitulate the pathology in the cell types affected in the patients and in animal models is encouraging and allows for the study of the affected cell types (VSMCs and MSCs) during differentiation and for drug screenings. 

iPSCs have also been used to test pharmacological treatments on mesodermal stem cells (derived from iPSCs). In particular, three main treatments offered to patients with premature aging syndromes, i.e., a farnesyltransferase inhibitor, an aminobisphosphonate and a statin together (zoledronate and pravastatin), and rapamycin (a macrolide antibiotic) [31]. These studies revealed the complexity of the modes of action of different drugs and highlighted the usefulness of iPSCs and their derived cells (in this case mesodermal stem cells) as critical and powerful tools for standardized, comparative pharmacological studies. Specifically, the robustness of this model has allowed researchers to develop a platform for pharmacological studies to schedule useful results that can be obtained by targeting specific pathological pathways. Moreover, the findings recently obtained (i.e., by [28]) provide new insights on the use of iPSCs for the development of pathological model system and can lead to novel therapeutic interventions for disorders that lack pre-clinical in vitro human models, such as HGPS [28]. 

Reprogramming of cells from centenarians or patients with HGPS, resets telomere size, gene expression profiles, and levels of oxidative stress, resulting in the generation of rejuvenated cells [27,30,32]. Serrano and Yamada groups have shown that cellular reprogramming to pluripotency, although associated with tumor development (e.g., teratoma formation), can be achieved in vivo in mice by the forced expression of the Yamanaka factors [33,34]. In addition, the partial reprogramming, in vitro, by transient expression of OSKM (*organic cation/carnitine transporter 4* or *OCT4, SRY (sex determining region Y) box 2* or *SOX2, Kruppel like factor 4* or *KLF4* and *myelocymatosis oncogene* or *MYC*) can induce dedifferentiated progenitor-like state [35,36]. Therefore, Belmonte hypothesized that partial in vivo reprogramming could slow or reverse the aging process and extend organismal lifespan. In fact, Belmonte’s group recently reported that cyclic in vivo induction of OSKM in a mouse model of premature aging improves age-associated phenotypes and extends lifespan [8]. This in vivo platform for the reprogramming of epigenetic markers may be used to better understand physiological aging, as well as the role of epigenetics during mammalian aging. The results obtained by Ocampo et al. [8] suggest that cyclic induction of OSKM following an “on and off” scheme may have the capacity to prevent or reset the accumulation of age-associated phenotypes. DNA methylation, post-translational modifications of histones, and chromatin remodeling are considered conserved hallmarks of aging [37,38] and, in fact, the technology of reprogramming somatic cells to pluripotency is based on a step-wise global epigenetic remodeling [37,39,40]. The rejuvenation of these epigenetic modifications has been demonstrated during cellular reprogramming to pluripotency in vitro [27,32,41,42], but the underlying mechanisms still lack a clear understanding. Moreover, since iPSCs can be differentiated into specific cell-types for replacement therapies, and the cells derived from autologous iPSCs should not cause immunological rejection upon transplantation, they represent a very powerful technology towards the success of personalized regenerative medicine [43]. The possibility to use iPSCs for human transplantation has significant promise, but this cellular model still deserves to be fully understood in their biological features, differentiation potential, and tumorigenic risk. In order to dissect the biological features of iPSCs and to understand whether iPSCs can age in vitro, Compagnucci’s group dissected several aspects of iPSCs aging. In particular, the study of the mitochondrial status [18], the nucleoskeletal properties [19] and a detailed ultrastructural analysis [44] allowed us to demonstrate that iPSCs maintained in culture in aerobic condition encounter a progressive aging process. Therefore, it is essential to deeply understand the biology of iPSCs in order to know the best culturing condition and potential problems arising from the expansion of iPSC lines prior to their use in regenerative medicine. Following this line, understanding the specific metabolism of iPSCs is fundamental to properly maintaining them and preventing aging.

### 4.2. Has the iPSCs Metabolism Been Elucidated at the Molecular Level?

Cellular metabolism is characterized by highly integrated life-sustaining biochemical reactions that use energy to keep the living state of the cells [45]. Each cell type is characterized by specific metabolic properties due to their niche, growth rate and physiological activity. Despite the fact that iPSCs do not exist in nature, they have a cellular metabolism similar to embryonic stem cells (being the cells that most closely resemble their physiological state). 

Despite the fact that metabolic mechanisms occurring during iPSC reprogramming remain mostly unknown, time-course analyses of different molecular features during iPSC generation have revealed a continuous shift in metabolic pathways. Together with the reorganization of the metabolic pathways during iPSC generation, mitochondrial remodeling has also been demonstrated. In fact, mitochondrial respiratory complexes are downregulated and the mitochondrial number is reduced, resulting in a mitochondrial network with major functional and structural changes [22,46,47]. Moreover, the suppression of succinate dehydrogenase complex subunit A (SDHA) by microRNA-31 (miR-31) contributed to the metabolic changes occurring during iPSC generation [48]. These metabolic switches are induced, partly, by modification of the epigenetic status of genes involved in glycolytic and oxidative phosphorylation or OXPHOS processes [49]. Specifically, this extensive metabolic reorganization paves the way toward a progressive transition from oxidative metabolism to glycolysis, as demonstrated by experiments performed during reprogramming, which demonstrated an augmented glycolytic rate and lactate production together with a decreased cellular respiration [47,49,50]. Hawkins et al. demonstrated that this early burst of OXPHOS and reactive oxygen species leads to an increase in nuclear factor erythroid 2 like 2 (NRF2) activity and the subsequent hypoxia inducible factor 1 alpha subunit or HIF-1α activation that leads to the metabolic switch during reprogramming [51]. Importantly, the physiologic niche of stem cells is anaerobic and this is an important aspect to consider when maintaining iPSCs in culture for both reprogramming studies [52,53] and iPSCs culture. In fact, the ability to recreate the correct stem cell niche in vitro is lacking and this hinders: (1) studying the biology of iPSCs, (2) using this technology to model human diseases and (3) expanding them for cell therapy. Therefore, it is necessary to deepen our understanding on the molecular determinants placed in the local niche and on the metabolic mechanisms controlling self-renewal versus differentiation. 

### 4.3. Are Precision Genome Editing Technologies Suitable for iPSCs Applications?

The recent advancements in molecular biology have allowed for the integrations of different technologies. One of these possibilities is to combine iPSC technology with precision genome editing. In particular, the clustered regularly interspaced short palindromic repeat (CRISPR)/Cas9 system has greatly paved the way for creating gene-targeted knock-out and knock-in animal models are created, thanks to the ease of guide RNA (gRNA) design and their delivery to one-cell embryo. Moreover, recent studies suggest the possibility to use the CRISPR/Cas9 system to successfully perform targeted gene therapy of human genetic diseases [54]. 

Despite the fact that recent studies of in vivo targeting animals lead to enormous expectations for the understanding and the cure of genetic pathologies, a number of challenges remain to be overcome. For example, optimization of the delivery of the CRISPR/Cas9 system according to the cell type of interest needs to be ameliorated [55].

Importantly, Liu et al. performed gene correction by LMNA-c-HDAdV (helper-dependent adenoviral vectors) in iPSCs derived from patients with *LMNA* mutations and observed that it maintains genetic and epigenetic integrity, reversing the disease-associated phenotypes [56].

One additional difficulty in the field of genome editing is the difficulty performing targeted transgene integration in non-diving cells (which comprise most adult tissues, especially in the nervous system). Importantly, a recent study by Suzuki et al. [57] demonstrated a homology-independent targeted integration (HITI) strategy (based on CRISPR/Cas9 technology) that allows for robust knock-in of both dividing and non-dividing cells in vitro and in vivo (i.e., in neurons of postnatal mammals). Moreover, Suzuki et al. used this technology to rescue the retinal degeneration in a rat model of retinitis pigmentosa, demonstrating its potential for in vivo targeted gene-replacement therapy [57]. 

In conclusion, iPSC gene editing offers the possibility to study human diseases using reliable models where iPSCs of both controls and patients differ only in the mutation of interest, which, therefore, represents the best control situation. In addition to this advantage, the combination of iPSC and gene editing technologies allows for correction of the mutation causing the disease and opens the possibility of transplanting the genetically corrected cells back into the patients. Although the goal of clinical application of iPSCs and gene editing for human therapies is still far away, each relevant discovery in this field continues to bring this closer to reality. 

## 5. Conclusions

The increase in life expectancy of modern times in industrialized countries has led to the increase in age-related disorders (i.e., cancer and type 2 diabetes). Because of the general interest in understanding the biology of aging, several research groups have focused their efforts toward this research area and this has allowed for the unveiling of some of the molecular pathways involved in the aging process (i.e., DNA damage, mitochondrial dysfunction and altered cell metabolism) [58]. However, despite these discoveries, the mechanistic networks driving aging are still unclear and studies related to aging are in need of multidisciplinary investigations deriving from different model systems (including worms, flies, mice, rats, and human iPSCs). 

Aging syndromes, and particularly premature aging syndromes, result in alterations of stem cells. In fact, many lines of evidence suggest that stem cell alterations have an important role in the pathogenesis of premature aging syndromes and a prompt discard of aged and/or dysfunctional stem cells is required to protect the patients. Unfortunately, effective pharmacological treatments to cure this disease do not exist. We hope that iPSCs offers a definitive solution to overcome the difficulties encountered in gathering the affected cell type without impairing the patients, and provides a more accurate alternative to the less reliable animal models used to validate the underpinnings of human stem cell biology. iPSCs offer a model able to recapitulate the steps in developmental biology that occur in human physiology. Specifically, they offer the possibility to study differentiation of iPSCs into disease relevant cell type, allowing our understanding of disease initiation and development. Moreover, the traditional view that metabolism is a developmental byproduct has been recently refuted and the link between metabolism and development/cell differentiation deserve future investigations [45].

**Figure 1:**
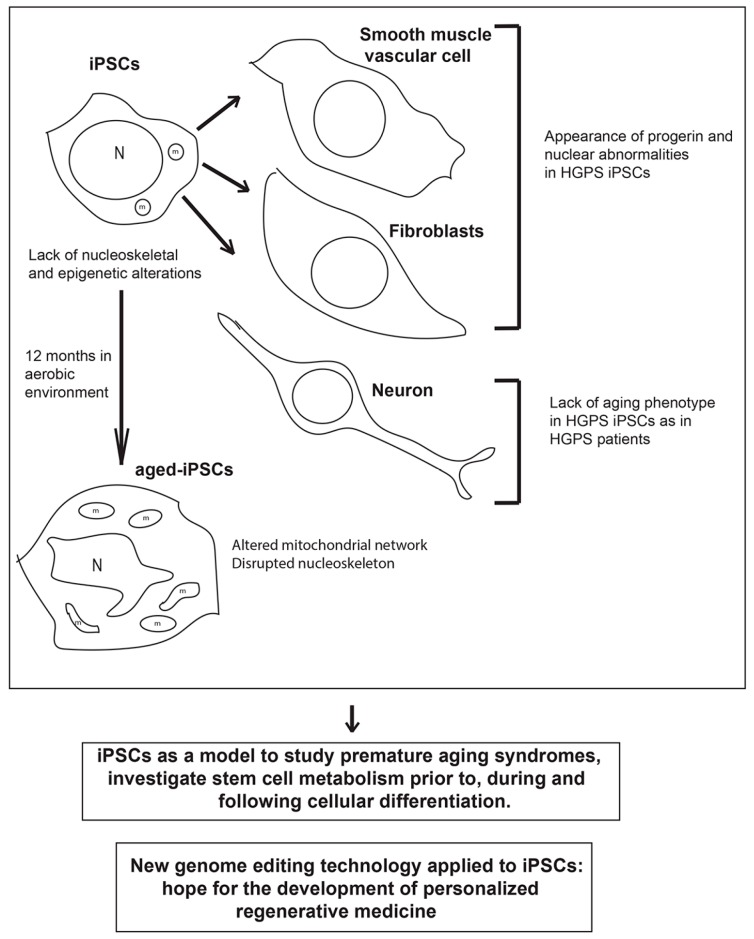
Schematic drawing of the main phenotypes observed in induced pluripotent stem cells (iPSCs) derived from patients with premature aging syndrome, which lack nucleoskeletal and epigenetic alterations, iPSC-derived differentiated cells (smooth muscle vascular cells and fibroblasts) which show increased PROGERIN levels and nuclear abnormalities, and neurons which lack the aged phenotype, and aged-iPSCs, which resemble some of the main features of the cells obtained from patients with premature aging syndromes. Below are reported two messages representing the main hopes and promises for the application of iPSC technology to efficiently treat premature aging disorders in the future. HGPS, Hutchinson-Gilford progeria syndrome; N, nucleus; m, mitochondrion.

In addition to the possibility of the use of iPSC-based models to dissect the initiation of specific human disorders and perform drug screenings, the recent advancements in the field of genome editing provides the possibility of an effective and feasible application of iPSCs for therapeutic intervention (Figure 1). In fact, models based on the iPSCs hold significant promise for the development of the personalized regenerative medicine. Nonetheless, before reaching the clinic, it is important to provide solid proof of concept of this strategy that acts on multiple pathways to treat multifaceted aspects of the disease.
